# Pyogenic Myositis: A Fulminant Presentation in an Immunocompromised Patient

**DOI:** 10.7759/cureus.77552

**Published:** 2025-01-16

**Authors:** João L Miranda, Francisca Carmo, Mariana Estrela, Raquel Moura, Filipe Breda

**Affiliations:** 1 Internal Medicine, Unidade Local de Saúde Gaia e Espinho, Vila Nova de Gaia, PRT

**Keywords:** fulminant presentation, immunosuppression, pyogenic myositis, septic shock, tropical myositis

## Abstract

Pyomyositis, a purulent infection of an individual muscle group, is an infrequent condition, mostly occurring in immunocompromised patients. It is often caused by Staphylococcus aureus and most of the time restricted to a single muscle group, with disseminated presentations being exceptionally rare. We present a case of severe pyogenic myositis with disseminated muscle involvement, presenting as a septic shock in an immunocompromised patient. The case highlights the importance of considering pyomyositis in the differential diagnosis of a patient with hematogenous dissemination of a pyogenic agent and sustained muscle complaints. A prompt radiological diagnosis and a multidisciplinary approach to treatment are often necessary for a good functional outcome.

## Introduction

Pyomyositis is defined as a purulent infection of individual muscle groups, caused mainly by Staphylococcus aureus (>90% of reported cases) [1], and most cases occur in immunocompromised patients [2]. Historically, it has been called tropical pyomyositis due to its geographical distribution and higher prevalence in underdeveloped countries, but cases have been reported all around the globe, particularly in patients with chronic HIV infection [1,2]. Typical symptoms are fever, tenderness of the affected muscle(s), and/or pain, and the lower limbs are the most common location of the purulent infection [3]; conversely, there are no specific laboratory findings, with creatine kinase levels being most often almost normal, which makes the diagnosis rely on imaging and microbiological findings [3,4]. Most patients have diseases limited to the affected muscle, but severe complications like septic shock and toxic shock syndrome have been reported [5]. We present a case of severe pyogenic myositis with multiple muscle involvement and presenting as a septic shock in an immunocompromised patient due to recent chemotherapy. 

## Case presentation

A 40-year-old male with a history of Burkitt lymphoma presented to the emergency department with fever, myalgias of the lower limbs, asthenia, and anorexia. His past medical history included stage IV Burkitt lymphoma (diagnosed one year prior and receiving chemotherapy treatment), a right upper-limb deep vein thrombosis associated with a peripheral inserted central catheter (managed with low-molecular-weight heparin anticoagulation), and anxiety. He had last received a chemotherapy cycle one week before, including the intrathecal administration of methotrexate and cytarabine. The patient reported a two-day history of lower limb myalgias and fever, with body temperature reaching 39.5ºC on the day of admission. On physical examination, he was pale and ill-appearing, but neurologically intact; his temperature was 38.2ºC, his heart rate was 102 beats per minute, his blood pressure was 80/46 mmHg, and he was breathing at 30 cycles per minute in ambient air, with peripheral O2 saturation of 100%. He presented with mild inflammatory signs in the left forearm, with pain on palpation and mobilization of most of the muscle groups in both upper and lower limbs. 

Initial laboratory studies are shown in Table 1.

**Table 1:** Laboratory studies results at the emergency department on admission.

Test (units)	Result	Reference range
Hemoglobin (g/dL)	7.8	14 - 18
Leukocytes (cells/uL)	90	3.800 - 10.600
Neutrophils (cells/uL)	< 100	> 2.200
Lymphocytes (cells/uL)	0	> 1.000
Platelets (cells/uL)	< 1.000	150.000 - 400.000
Creatine kinase (U/L)	628	
C-reactive protein (mg/L)	395.3	0 - 5
Electrolytes, liver, and renal function	Normal	- - -

The patient had severe pancytopenia and an important elevation in inflammatory markers, with a slightly raised creatine kinase (around three times the upper normal limit). The chest X-ray was normal, and an abdominal and pelvic CT scan was also performed, which showed no relevant abnormalities. Blood cultures were drawn, and broad-spectrum antibiotic coverage with piperacillin/tazobactam, vancomycin, and micafungin was initiated, and the patient was admitted to the intensive care unit (ICU) with septic shock and associated multiorgan dysfunction. 

**Figure 1:**
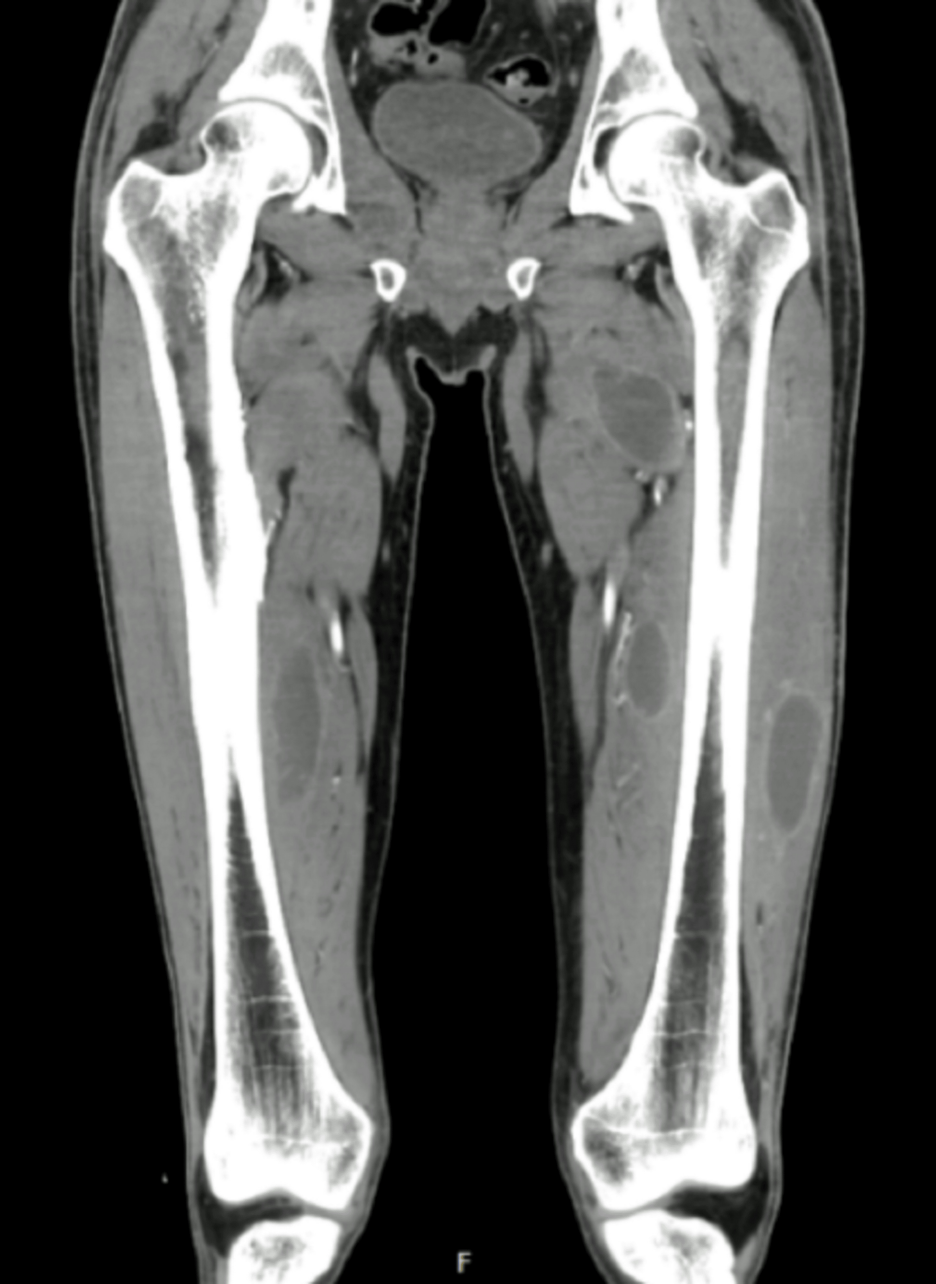
Contrast-enhanced computed tomography of the lower limbs showing multiple collections across several muscle groups of the thighs (coronal view).

**Figure 2:**
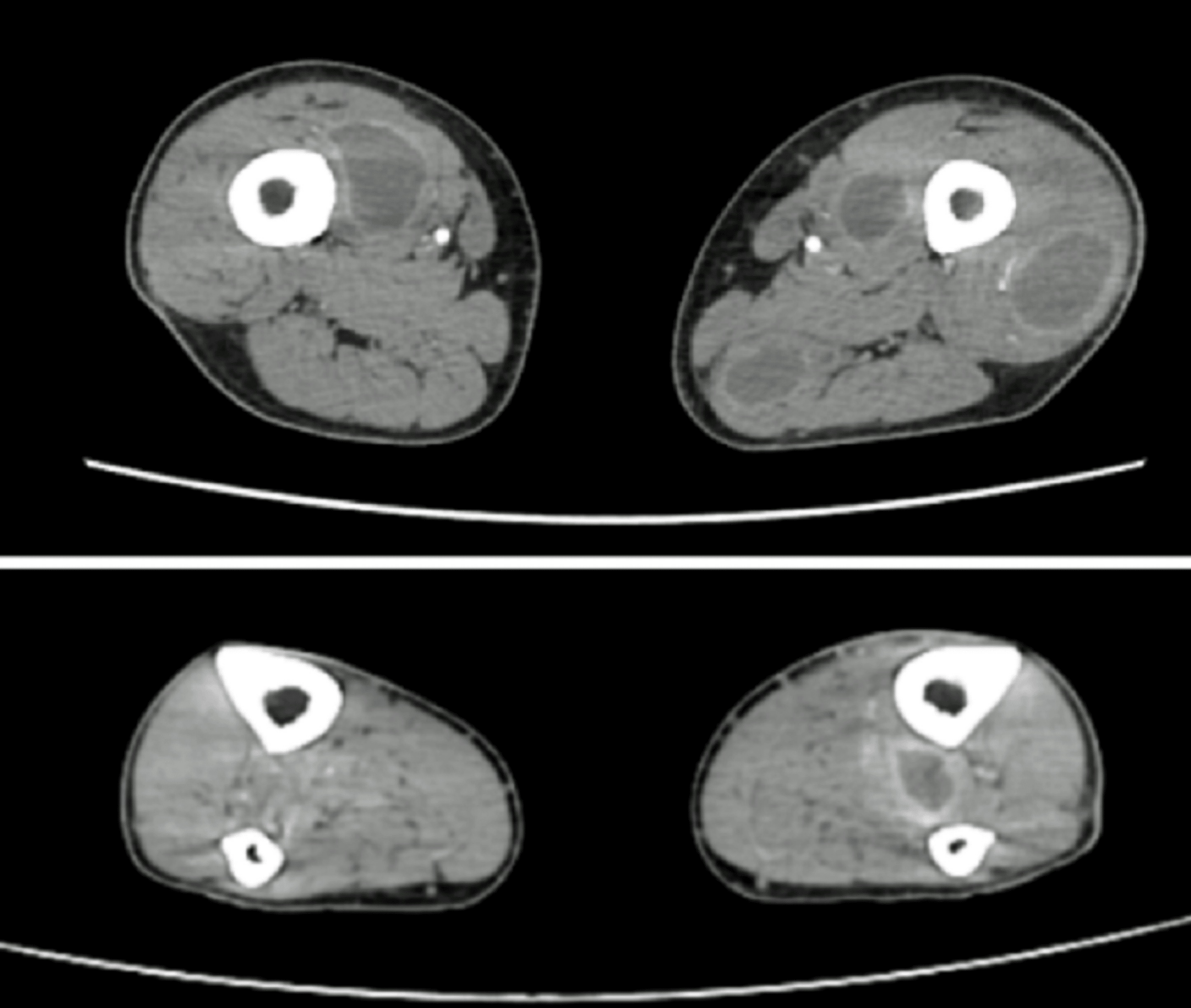
Contrast-enhanced computed tomography of the lower limbs showing multiple collections across several muscle groups (axial view) at two different levels: thighs (above) and legs (below).

On the following day, blood cultures came back positive for methicillin-susceptible Staphylococcus aureus (MSSA), and antibiotic therapy was de-escalated to flucloxacillin. After sustained clinical improvement and resolution of organ dysfunctions, the patient was discharged from the ICU after four days and admitted to the internal medicine ward, where he continued to report myalgias in the lower limbs and difficulty walking. A hardened area was identified on the lateral aspect of his left thigh, and musculoskeletal ultrasound revealed a heterogeneous collection suggestive of liquefaction (48 x 18 x 127 mm) in the vastus lateralis muscle. A computed tomography of the lower limbs was performed, which revealed numerous collections across almost every muscle group in both lower limbs, as depicted in Figures 1, 2.

Percutaneous drainage of the collections by interventional radiology was unsuccessful, requiring a multidisciplinary surgical approach to drain the major lesions. Purulent material of the drainage was also sent for culture and confirmed MSSA as the causative organism. 

Intravenous flucloxacillin was continued until there was radiographic evidence of improvement in the abscesses. At that point, he was switched to oral sulfamethoxazole + trimethoprim (due to an easier dosing scheme) and discharged home with close outpatient follow-up. He was kept on antibiotic therapy for a total of 16 weeks, alongside motor rehabilitation, recovering all of his previous autonomy. Control CT scan of the lower limbs after six months of admission showed no signs of organized collections or other relevant abnormalities.

## Discussion

Pyogenic myositis is a rare condition that was once considered to exist only in tropical and underdeveloped areas [2]. However, it is increasingly recognized among immunocompromised patients, and every year new cases are described all over the world [6]. The disease can occur in all age groups but is more common in children and young adults (20-45 years), with a slight predominance of male patients [7]. It is typically an indolent disease and localized to a single muscle group, and fulminant presentations like the one described are exceptionally rare, with only a few cases reported worldwide [8,9].

Early imaging exams and microbiological cultures, along with the institution of appropriate antibiotic therapy and drainage of significant purulent collections, are essential for a favorable outcome. Early rehabilitation should also be offered to these patients, as muscular pain can be an obstacle to functional improvement. 

## Conclusions

Given its rarity, pyogenic myositis is frequently overlooked in initial differential diagnoses; therefore, it is essential that patient complaints and objective findings are integrated to establish an accurate diagnosis.

Furthermore, considering the increasing global prevalence of chronically immunosuppressed patients, pyogenic myositis should be a diagnostic consideration in immunocompromised individuals presenting with fever and muscular complaints. A delay in diagnosis and appropriate treatment can have severe and potentially irreversible consequences. A high level of clinical suspicion, imaging studies, and laboratory confirmation are essential to ensure prompt initiation of therapy and a favorable outcome for the patient.
